# Detrimental and Neutral Effects of a Wild Grass-Fungal Endophyte Symbiotum on Insect Preference and Performance

**DOI:** 10.1673/031.011.7701

**Published:** 2011-06-29

**Authors:** Stephen L. Clement, Jinguo Hu, Alan V. Stewart, Bingrui Wang, Leslie R. Elberson

**Affiliations:** ^1^ USDA, ARS Plant Germplasm Introduction and Testing Research Unit, 59 Johnson Hall, Washington State University, Pullman, WA 99164-6402, USA; ^2^ PGG Wrightson Seeds, PO Box 175, Lincoln, New Zealand; ^3^ Huazhong Agricultural University, Wuhan, China

**Keywords:** Aphididae, Chrysomelidae, grass endophyte, insect herbivory, host-plant resistance, *Oulema melanopus*, *Rhopalosiphum padi*, wild grass *Phleum alpinum*

## Abstract

Seed-borne *Epichloë/Neotyphodium* Glenn, Bacon, Hanlin (Ascomycota: Hypocreales: Clavicipitaceae) fungal endophytes in temperate grasses can provide protection against insect attack with the degree of host resistance related to the grass—endophyte symbiotum and the insect species involved in an interaction. Few experimental studies with wild grass—endophyte symbiota, compared to endophyte-infected agricultural grasses, have tested for anti-insect benefits, let alone for resistance against more than one insect species. This study quantified the preference and performance of the bird cherry oat-aphid, *Rhopalosiphum padi* (L.) (Hemiptera: Aphididae) and the cereal leaf beetle, *Oulema melanopus* (L.) (Coleoptera: Chrysomelidae), two important pests of forage and cereal grasses, on *Neotyphodium*-infected (E+) and uninfected (E-) plants of the wild grass Alpine timothy, *Phleum alpinum* L. (Poales: Poaceae). The experiments tested for both constitutive and wound-induced resistance in E+ plants to characterize possible plasticity of defense responses by a wild E+ grass. The aphid, *R. padi* preferred E- over E+ test plants in choice experiments and E+ undamaged test plants constitutively expressed antibiosis resistance to this aphid by suppressing population growth. Prior damage of E+ test plants did not induce higher levels of resistance to *R. padi.* By contrast, the beetle, *O. melanopus* showed no preference for E+ or E- test plants and endophyte infection did not adversely affect the survival and development of larvae. These results extend the phenomenon of variable effects of E+ wild grasses on the preference and performance of phytophagous insects. The wild grass— *Neotyphodium* symbiotum in this study broadens the number of wild E+ grasses available for expanded explorations into the effects of endophyte metabolites on insect herbivory.

## Introduction

Microbial associates of temperate grasses in the form of seed-borne *Epichloël Neotyphodium* (Ascomycota: Hypocreales: Clavicipitaceae) fungal endophytes can influence host grass suitability for insect herbivores by deterrence or by reduction in insect survival and development via the production of specific alkaloids (Clay 1988; Clement et al. 1994; Lane et al. 2000; Popay 2009). The first reports linking grass endophytes with host resistance to insects appeared in the 1980s with documented field resistance of *Neotyphodium*-infected perennial ryegrass, *Lolium perenne,* to the Argentine stem weevil, *Listronotus bonariensis,* in New Zealand (Prestidge et al. 1982; Stewart 1985), and to the sod webworm, *Crambus* sp., in the U.S. (Funk et al. 1983). Since the 1980s, much has been learned about factors that influence the outcome of grass—endophyte— insect interactions. For example, the expression and type of insect resistance (antixenosis, antibiosis) may be affected by host grass species/genotype, endophyte strain (including associated alkaloid profile), and insect species/genotype involved in a given interaction (Breen 1994; Clement et al. 1994; Clement et al. 2005; Afkhami and Rudgers 2009; Bieri et al. 2009; Cheplick and Faeth 2009; Popay 2009; Crawford et al. 2010). Today, the number of herbivorous insects that have been reported to be negatively affected by endophyte-infected (E+) grasses has grown to over 40 species (Kuldau and Bacon 2008; Popay 2009). However, endophyte infection does not always confer host grass resistance to insects (Kirfman et al. 1986; Lewis and Clements 1986; Lopez et al. 1995; Saikkonen et al. 1999).

The bioprotective alkaloids in grass— endophyte symbiota are generally grouped as ergot alkaloids, indole diterpenes (generally lolitrems), pyrrolizidine lolines, and pyrrolopyrazines. All of these alkaloid classes have anti-insect activity (Siegel et al. 1990; Siegel and Bush 1996; Lane et al. 2000; Schardl et al. 2007; Potter et al. 2008). However, only pyrrolizidine loline derivatives (*N*-formyl loline, *N*-acetyl loline, *N*-acetyl norloline) and peramine (pyrrolopyrazine) are widely viewed as important in the insect resistance of E+ grasses because, unlike ergot and lolitrem alkaloids in E+ grasses, they have no known toxic effects on vertebrates (Bush et al. 1997; Lane et al. 2000). One approach to overcoming animal toxicosis problems is to plant grass cultivars harboring naturally occurring *Neotyphodium* strains that do not produce mammalian toxins (such as ergot and lolitrem alkaloids), but still produce the necessary metabolites for insect resistance and other ecological benefits (Latch 1997; Bouton 2009). Other endophyte metabolites (i.e., epoxy-janthitrems) may have bioprotective properties (Popay and Wyatt 1995; Ball et al. 2006), with more to be discovered (Lane et al. 2000).

Continued commercial development of new grass—endophyte combinations for insect resistance and improved agronomic persistence is contingent on the availability of a diverse pool of novel (nontoxic to mammals) *Neotyphodium* strains in wild grasses (Clement et al. 1994; Clement et al. 2008). Therefore, it is important to discover and document the existence of diverse wild grass—endophyte symbiota and to characterize the responses of globally important graminoid pests to these associations. To date, most endophyte studies have tested insect responses to agriculturally important grasses such as tall fescue, *Lolium arundinaceum,* and perennial ryegrass (Saikkonen et al. 2006; Crawford et al. 2010). The discovery of a *Neotyphodium*-infected wild *Phleum* grass from Argentina (see Results) provided an opportunity to assess the preference and performance of important graminoid insect pests on E+ and uninfected (E-) plants of a wild temperate grass. While the exact identity of the *Neotyphodium* isolate in this grass (accession W6 23409) is unknown, Gentile et al. (2005) provided evidence of endophyte diversity in Argentine Alpine timothy, *Phleum alpinum* L. (Poales: Poaceae) (listed as *P. commutatum* Gaudin) when one isolate (Phc755) fit the description of *N. tembladerae* Cabrai and White and one (Phc682) did not.

The objective of this study was to experimentally quantify the host preference and performance of the bird cherry oat-aphid, *Rhopalosiphum padi* (L.) (Hemiptera: Aphididae) and cereal leaf beetle, *Oulema melanopus* (L.) (Coleoptera: Chrysomelidae) on E+ and E- wild *P. alpinum* plants. Because the aphid *R. padi* has been used to draw conclusions about the importance of constitutive and inducible resistance in E+ grasses (Bultman and Murphy 2000; Bultman and Bell 2003; Bultman et al. 2004; Sullivan et al. 2007), the *R. padi* experiments in this study tested for the expression of both resistance types by recording aphid responses to damaged and undamaged *P. alpinum* test plants. Although *R. padi* is not a recorded pest of forage grasses in the genus *Phleum,* this aphid is an important vector of barley yellow dwarf virus (BYDV) (Guy et al. 1987; Power et al. 1991). More knowledge about the antixenotic properties of E+ grasses will determine the potential for using endophyte infection to repel host-seeking *R. padi* with the potential to transmit BYDV (Lehtonen et al. 2006). Cultivated timothy grass, *Phleum pratense* L., and alpine timothy, *P. alpinum* (PI 619539), are recorded feeding hosts of *O. melanopus* (Wilson and Shade 1966; Clement and Elberson 2010); however, this beetle is best known as an important pest of cereal crops in Europe and North America, particularly wheat, *Triticum aestivum,* oats, *Avena sativa,* and barley, *Hordeum vulgare* (CAB International 2002).

## Materials and Methods

### Plants, insects, and *Neotyphodium* detection

The germplasm accession W6 23409 was evaluated with seed originally collected from wild plants in Argentina (Province of Santa Cruz) and stored in the seed bank at the USDA, ARS Western Regional Plant Introduction Station, Pullman, Washington USA. This accession is identified as *P. commutatum* in the GRIN database (Genetic Resources Information Network: http://www.ars-grin.gov/npgs) of the U.S. National Plant Germplasm System. However, *P. alpinum* (= W6 23409) is used in this paper because *P. commutatum* is a synonym of this species (Soreng et al. 2003), which has the widest global distribution of any *Phleum* species (Stewart et al. 2009).

Seed to grow aphid and beetle E+ and E- test plants was produced by four E+ (seed bulked) and five E- plants (seed bulked) of *P. alpinum* (18–24 months old). The aphid test plants were 10–11 weeks-old and selected on the basis of having equivalent amounts of plant material (2–3 tillers with 12–14 leaves and no senescing tissue). The beetle experiments were conducted with 14–15 week-old test plants that had equivalent amounts of tiller and leafy material (5–6 tillers, 25–30 leaves, and no senescing tissue). In addition, four 5-year-old tall fescue plants (cv. Kentucky 31), two previously identified as *Neotyphodium*infected and two endophyte-free (Clement 2009), served as *Neotyphodium* (E+, E-) controls for PCR (see below). All plants were maintained in a glasshouse (13–33°C; 10–16 hours of natural light) where they were watered as needed and fertilized bi-weekly with a soluble 20-20-20 fertilizer (0.6g/L).

*R. padi* for experiments were obtained from a laboratory colony reared on wheat, *T. aestivum* (cv. Stevens), in a growth chamber (21 ± 2°C, 14:10 L:D). The wheat plants in 10-cm pots were replaced every 14 days to avoid aphid overcrowding. This colony was initiated with progeny of 12 alates from a colony maintained by the Department of Entomology, Washington State University, Pullman, Washington, in April 2003. Adults of *O. melanopus* were collected in April 2010 in a wheat field near Connell, Washington (46°42′N, 118°51′W) and maintained on potted barley, *H. vulgare* (cv. UC 937), plants in laboratory (21–23° C) cages (39h × 33w × 43d cm). This caged population supplied adult beetles and neonate larvae for experiments (described below). Neonate larvae were obtained from eggs that were incubated on moistened filter paper in glass Petri dishes (21–23° C) until hatch.

Two methods were used to detect *Neotyphodium* endophyte in plants. The first was fungal isolation on potato dextrose agar supplemented with streptomycin sulfate and tetracycline hydrochloride (50 µg each per ml) for suppression of bacteria. Following procedures in Clement et al. (2001), basal stem sections (∼1 cm in length) from 1–2 tillers per plant were surface-disinfected and placed on potato dextrose agar in sealed polystyrene Petri dishes and incubated in a laboratory (complete darkness, room temperature). Petri dishes were examined for mycelial growth from plant tissue at 2–3 day intervals for 45 days. A plant was scored E- if *Neotyphodium* mycelia did not appear during this period of time. The fungus was confirmed as *Neotyphodium* from published descriptions of colonies on agar (Latch et al. 1984; White and Morgan-Jones 1987), although the exact identity of this endophyte (strain/species) has not been established. This isolation method determined the E- or E+ status of the nine *P. alpinum* ‘seed source plants’ and all aphid and beetle test plants.

For PCR, total plant DNA was extracted from grass tiller and leaf sheath tissue using the DNeasy 96 Plant Kit (Qiagen, www.qiagen.com) according to the manufacturer's instructions. A basal section (∼1 cm in length) of one new-growth tiller per plant was removed for DNA preparation. Resulting DNA samples were quantified with a microplate flourometer Fluoroskan Ascent FL (Thermo Systems, www.thermoscientific.com) and adjusted to a concentration of 20 ng/µl for PCR amplification. This method determined the *Neotyphodium* infection status of six 2-month-old *P. alpinum* plants. In addition, PCR confirmed the presence or absence of *Neotyphodium* endophytes in four ‘seed source plants’ and four Kentucky-31 tall fescue plants (see Results).

Amplification was carried out using *Neotyphodium* spp.-specific primer pairs tef1-exon1d-1 (5′- GGG TAA GGA CGA AAA GAC TCA -3′) and tef1-exon5u-1 (5′- CGG CAG CGA TAA TCA GGA TAG -3′) targeting translation elongation factor 1-alpha *(tef1),* and tub2-exon1d-1 (5′- GAG AAA ATG CGT GAG ATT GT -3′) and tub2-exon4u-2 (5′- GTT TCG TCC GAG TTC TCG AC -3′) targeting the tubulin 2 gene *(tub2)* (Moon et al. 2002). Amplification of *Neotyphodium* fragments was achieved in a total volume of 20 µl containing the following components: 3 µl of 20 ng/µl DNA, 0.8 µl of 50 mM MgCl_2_, 1.6 µl of 2.5 mM of dNTPs, 3 µl of 2 µM of each primer, 2 µl of 10X PCR buffer, 0.4 µl of Hot-Start AccuSure polymerase 5 units/µl (Bioline USA Inc., www.bioline.com), and 6.2 µl of H_2_O. A 96-Well GeneAmp® PCR System 9700 (Applied Biosystems, www.appliedbiosystems.com) was programmed for 9 minutes at 94° C for polymerase activation, followed by 40 cycles of 94° C, 1 minutes, 60° C 1 minutes and 72° C 1 minutes, and a 5 minute incubation at 72° C for final extension. Amplified products were separated by electrophoresis in 1.5% agarose gels alone with the DNA size standard HyperLadder I (Bioline) at 100 volt for 1 hour. The gel was stained with 0.2 µg/ml ethidium bromide and pictured with a Molecular Image Gel Doc™ XR System (BIO-RAD, www.bio-rad.com).

### Aphid experiments

The endophyte status of aphid test plants not subjected to prior damage (‘undamaged’) was determined 4–5 weeks (tiller sections on potato dextrose agar) after experiments were completed. ‘Damaged test plants’ were artificially damaged 4 days before experiments began by cutting-off the largest tiller (4 mm above the soil) of each plant and leaving the remaining 2–3 tillers undamaged. These damaged test plants were scored for endophyte status by placing sections from clipped tillers on potato dextrose agar. This method of tiller clipping to simulate a wound-inducible response by W6 23409 is modeled after the controlled *Lolium-* and *Festuca*-insect experiments of Bultman and Murphy (2000) and Bultman et al. (2004).

Two paired-choice experiments recorded the preference responses of *R. padi* to E+ and E- plants that were either undamaged (experiment 1) or damaged as described above (experiment 2). Each experiment had six large acrylic cages (51 × 51 × 51 cm) randomly positioned on a glasshouse bench (15.6–26.7° C; ∼13 hours of natural light). In each cage, one E+ and one E- potted plant were placed in the bottom half of a seed germination box (11 × 11 cm with 3 cm rim) and angled towards each other (∼30°, pot rims touching) to entangle tillers and leaves from the two test plants. The leaning plants were held in place by the rim of each germination box. Four wheat leaves with 240 apterous aphids from the laboratory colony were draped over entangled plant material in each cage. As wheat leaves dried, aphids dispersed to the tillers and leaves of test plants. The total number of aphids on each plant was recorded after 48 hours.

Aphid population growth was quantified on four groups of *P. alpinum* test plants: 11 E+ undamaged, 8 E- undamaged, 7 E+ damaged, and 6 E- damaged plants (32 plants). Each potted plant was infested with 50 adult apterous aphids and encircled by a clear plastic vented tubular cage (36 mm diam. by 30 cm tall) that was tightly inserted into the soil. Each cage was capped with nylon organdy screen. The aphid-infested plants were randomly arranged in a growth chamber (21 ± 2° C; 14:10 L:D) and the total the number of aphids on each plant was recorded after 14 days.

### Beetle experiments

A paired-choice experiment recorded the feeding and oviposition preferences of adult *O. melanopus* for E+ and E- test plants. Mating adults (3♀ and 3♂) were transferred from the caged laboratory population to glass vials, starved for 4 hours, then placed on a Petri dish lid on the top of an inverted 10-cm pot placed between one E+ and one E- potted plant (10 cm) in an acrylic cage (51 × 51 × 51 cm). There were six replicate cages randomly positioned on a greenhouse (15.6–26.7° C; ∼13 h of natural light) bench. After 24 hours, all adults were removed and plants were taken to a laboratory to measure the length of adult feeding scars on leaves and to count eggs on each plant.

A second beetle experiment recorded larval development (number of days for neonate larvae to reach the fourth instar) and survival on E+ and E- test plants in a growth chamber experiment (21 ± 2° C; 16:8 L:D). Six E+ and 6 E- plants of *P. alpinum* were arranged in accordance with a completely randomized design and positioned in the large growth chamber so leaves from different plants would not touch. This approach prevented plant-toplant movement by larvae, as revealed by a pilot study and published research (Clement and Elberson 2010). Each plant was infested with 3 neonates (2–8 hours post-egg hatch) transferred with a fine-hair brush from hatching eggs incubated in glass Petri dishes. Plants were observed daily to record the number of larvae surviving to the fourth instar and the number of days for each neonate larva to reach this stage on each test plant. Instar determinations were made by measuring head capsule widths (Hoxie and Wellso 1974) with a dissecting microscope.

### Statistical analyses

The observed frequency of *R. padi* on E+ and E- plants in preference experiments was compared with an expected 50:50 ratio using a replicated G test (Sokal and Rohlf 1981), whereas *O. melanopus* feeding preference data were analyzed by a two-tailed *t*-test (*P* < 0.05) (SAS Institute Inc. 2006). Data from the *R. padi* population growth experiment were analyzed by two-way ANOVA to assess the effects of interactions (PROC GLM; SAS Institute Inc. 2006). Data from the *O. melanopus* larval development experiment were analyzed by one-way ANOVA. Analyses of raw data from the aphid and beetle antibiosis experiments met the normality assumptions of ANOVA according to the Shapiro-Wilk *W* test (SAS Institute Inc. 2006). Too few eggs were laid by *O. melanopus* in the preference experiment to permit statistical analysis.

## Results

### PCR and amplification products

**Figure 1.**
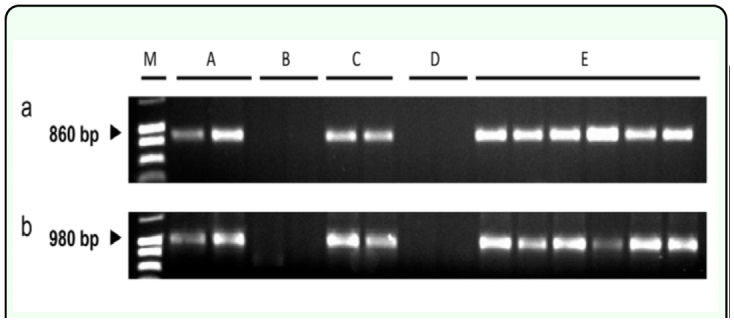
Amplification *of tef1* (panel a) and *tub2* (panel b) genes after polymerase chain reaction (PCR) with primer pairs tef1exon1d-1/ tef1-exon5u-1 and tub2-exon1d-1/ tub2-exon4u-2 and DNA extracted from *Neotyphodium*-infected (E+) and *Neotyphodium*-free (E-) tissue from tall fescue (Kentucky-31) and *Phleum alpinum* (W6 23409) plants. Letters above panel a and lanes indicate: (M) molecular weight standards (1000, 800 and 600 base pairs) in leftmost lane; (A) two 5-year-old E+ tall fescue plants; (B) two 5year-old E- tall fescue plants; (C) two 24-month-old E+ *P. alpinum* plants; (D) two 24-month-old E- *P. alpinum* plants; and (E) six 2-month-old E+ *P. alpinum* plants. High quality figures are available online.

Each of the two primer pairs yielded amplifications of target fragments from genes *tub2* and *tef1.* The approximate sizes of the amplified products were 980bp for *tub2* and 860bp for *tef1,* as expected for *Neotyphodium* isolates (Moon et al. 2002). *Neotyphodium-*specific bands were evident for eight *P. alpinum* (W6 23409) plants of different ages (2 years old (n = 2) and 9 weeks old (n = 6)) and, as expected, for two *Neotyphodium*infected tall fescue plants (5 years old). The diagnostic amplification products were not detected with samples from two E- tall fescue and two E- *P. alpinum* plants (Figure 1).

### Aphid experiments

**Figure 2.**
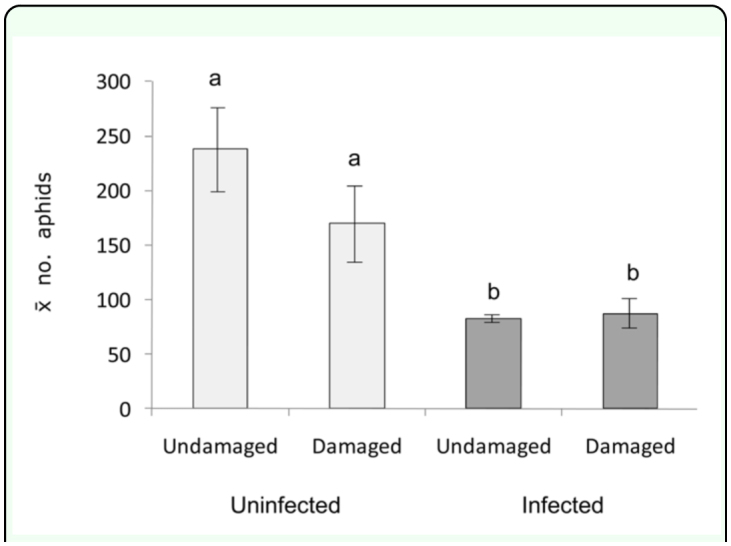
Mean numbers of *Rhopalosiphum padi* aphids on undamaged and damaged *Phleum alpinum* plants with (E+) and without (E-) *Neotyphodium* endophyte. Histograms with different letters above them are significantly different (ANOVA and LSD a-posteriori test, *P* < 0.0001). Error bars ± SEM. High quality figures are available online.

In preference experiments, variable numbers of *R. padi* (apterous + alates) settled on plants after 48 hours, exemplified by results of experiment 1 (Table 1). Fewer than 10 alates were counted in each cage after 48 hours. Aphids consistently preferred undamaged Eplants over undamaged E+ plants in all replicates (significant pooled value, *p* < 0.001). However, a significant heterogeneity value (*p* < 0.001) indicates this preference was not uniform in magnitude across all replicates (Table 1). Aphids also preferred damaged Eplants over damaged E+ plants in experiment 2 (data not shown) (significant pooled value of 7.24, *p* < 0.01), although a significant heterogeneity value of 50.78 (*p* < 0.001) indicates that aphids did not prefer one plant type (E- or E+) over the other in all replicates. In the population growth experiment, the effect of *Neotyphodium* infection of damaged and undamaged plants on aphid densities was highly significant (Figure 2). Mean aphid densities were significantly lower (F_1, 31_ = 24.43, *p* < 0.001) on undamaged and damaged E+ plants (mean of 84.5 aphids per plant) compared to densities on undamaged and damaged E- plants (mean of 209.21 aphids per plant). Apterous aphids were the vast majority of aphids in each cage (0 to 5 alates per cage). There was no effect of plant damage on mean aphid densities (F_1, 31_ = 1.70, *p* > 0.2030) (means of 125.77 and 148.16 aphids per damaged and undamaged plant, respectively). Moreover, the interaction between the main effects of endophyte infection and plant damage was not a significant source of variation on aphid densities (F_1, 31_ = 2.26, *p* > 0.1443).

**Table 1.**
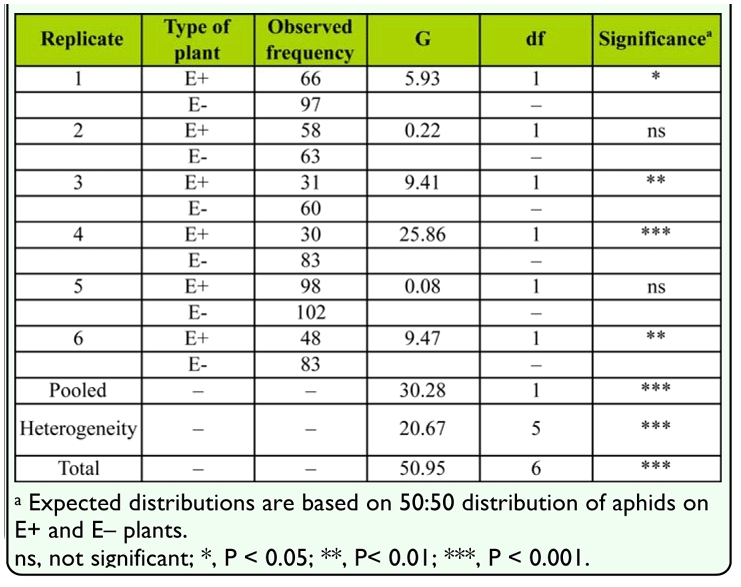
Distribution of *Rhopalosiphum padi* on undamaged endophyte-infected (E+) and uninfected (E-) plants of *Phleum alpinum*

### Beetle experiments

In the feeding preference experiment, *O. melanopus* adults showed no preference for E(feeding scar length of 74.5 ± 19.7 mm, mean ± SEM) or E+ (feeding scar length of 97.3 ± 40.5 mm) plants of *P. alpinum* (*t* = 0.4471, *p* = 0.6735). No beetles died and females laid a total of 14 and 6 eggs on E+ and E- plants, respectively, during this 24 hour experiment. In the antibiosis experiment, larval development periods (neonate to 4^th^ instar) were statistically similar (*F*_1,34_ = 0.02, *p* > 0.8878) on E- (10.94 ± 0.30 days, and E+ (10.89 ± 0.25 days) test plants, with no mortality.

## Discussion

The results herein further document variable effects of E+ grasses on the preference and performance of phytophagous insects (Breen 1994; Clement et al. 1994; Saikkonen et al. 1999; Afkhami and Rudgers 2009; Clement 2009; Popay 2009) by showing that two graminoid pests responded differently to *Neotyphodium* infection of wild *P. alpinum.* In 48 hour preference experiments, *R. padi* preferred E- over E+ test plants. However, numbers of aphids on E+ test plants were sufficiently high to cast doubt on the ability of E+ *P. alpinum* to repel all host-seeking *R. padi* with the potential to transmit BYDV. This aphid can transmit BYDV to cereal host plants in < 18 hours (Power et al. 1991). Of note, however, is that incidental infestations of *R. padi* were repeatedly observed only on E- potted plants among E+ potted plants on a greenhouse bench (SL Clement, personal observations); thus, E+ W6 23409 may exhibit strong aphid repellent properties under some conditions. Loline alkaloid production by E+ tall fescue and meadow ryegrass, *Lolium pratense* (= meadow fescue, *Festuca pratensis*), has been associated with *R. padi* deterrence (Wilkinson et al. 2000), leading to suggestions that grass—endophyte symbiota producing lolines could potentially be used to influence BYDV transmission in agricultural settings (Lehtonen et al. 2006).

Growth of *R. padi* populations was significantly suppressed on E+ *P. alpinum* plants compared to growth on E- test plants. Moreover, E+ undamaged plants of *P. alpinum* constitutively expressed this antibiosis resistance to *R. padi. Neotyphodium* infection provided constitutive defenses in tall fescue against *R. padi* in prior studies (Eichenseer et al. 1991; Bultman and Bell 2003). Prior studies also showed that damaged E+ plants of tall fescue (Bultman et al. 2004; Sullivan et al. 2007) and *Glyceria striata* (Gonthier et al. 2008) were more resistant to *R. padi* than undamaged E+ tests plants of both grasses. By contrast, our study recorded low but equivalent numbers of *R. padi* on both undamaged and damaged E+ *P. alpinum,* thus showing that prior damage of E+ plants of a wild grass did not induce higher levels of resistance to *R. padi.* Finally, damaged Eplants were not significantly more susceptible to *R. padi* than undamaged E- test plants. In contrast to our result, Bultman et al. (2004) and Sullivan et al. (2007) found that prior damage rendered E- tall fescue more susceptible to *R. padi.* Clearly, there is much more to learn about the herbivore defense strategies employed by different grass—endophyte symbiota, including the importance of both constitutive and induced plant responses in mediating interactions with insect herbivores.

In contrast to the experimental results with *R. padi,* there was no evidience that endophyte infection of *P. alpinum* (W6 23409) provided defense against the beetle *O. melanopus.* The beetle showed no preference for E+ or E- test plants, and endophyte infection did not adversely affect the survival and development of larvae. In other *O. melanopus* studies involving different grass—endophyte symbiota, significantly more larvae survived on E- than on E+ Kentucky-31 tall fescue plants (Clement et al. 2009), whereas similar mortality rates were recorded on both E- and E+ plants of a *P. alpinum* accession from Russia (PI 619539) (Clement and Elberson 2010).

This study was conducted in controlled environments and with plant fertilization, soil moisture, and temperature conditions optimal for plant growth, all factors that could potentially influence the outcome of grass— endophyte—insect interactions (Bultman and Bell 2003; Lehtonen et al. 2005; Cheplick and Faeth 2009). Therefore, the results herein may not reflect patterns in the field where endophyte effects on herbivores might differ from those recorded in laboratory tests (Krauss et al. 2007). Notwithstanding these conditions and potential limitations, the results indicate that *Neotyphodum* infection can mediate strong constitutive responses by a wild grass attacked by a pest aphid.

In conclusion, the different responses exhibited by *R. padi* and *O. melanopus* on a grass-endophyte symbiotium in this study are likely based in the types of endophyte metabolites and concentrations produced by this symbiotum. This study broadens the base of wild grass—endophyte symbiota for expanded exploration into the effects of endophyte metabolites on insect herbivory.

## Abbreviations

**BYDV**,: barley yellow dwarf virus;
**E+**,: endophyte infected;
**E-**,: endophyte uninfected
